# Predicting biological system objectives de novo from internal state measurements

**DOI:** 10.1186/1471-2105-9-43

**Published:** 2008-01-24

**Authors:** Erwin P Gianchandani, Matthew A Oberhardt, Anthony P Burgard, Costas D Maranas, Jason A Papin

**Affiliations:** 1 Department of Biomedical Engineering University of Virginia Box 800759, Health System Charlottesville, VA 22908 USA; 2 Genomatica, Inc. San Diego, CA 92121 USA; 3 Department of Chemical Engineering The Pennsylvania State University University Park, PA 16802 USA

## Abstract

**Background:**

Optimization theory has been applied to complex biological systems to interrogate network properties and develop and refine metabolic engineering strategies. For example, methods are emerging to engineer cells to optimally produce byproducts of commercial value, such as bioethanol, as well as molecular compounds for disease therapy. Flux balance analysis (FBA) is an optimization framework that aids in this interrogation by generating predictions of optimal flux distributions in cellular networks. Critical features of FBA are the definition of a biologically relevant objective function (e.g., maximizing the rate of synthesis of biomass, a unit of measurement of cellular growth) and the subsequent application of linear programming (LP) to identify fluxes through a reaction network. Despite the success of FBA, a central remaining challenge is the definition of a network objective with biological meaning.

**Results:**

We present a novel method called **Biological Objective Solution Search (BOSS) **for the inference of an objective function of a biological system from its underlying network stoichiometry as well as experimentally-measured state variables. Specifically, **BOSS **identifies a system objective by defining a putative stoichiometric "objective reaction," adding this reaction to the existing set of stoichiometric constraints arising from known interactions within a network, and maximizing the putative objective reaction via LP, all the while minimizing the difference between the resultant *in silico *flux distribution and available experimental (e.g., isotopomer) flux data. This new approach allows for discovery of objectives with previously unknown stoichiometry, thus extending the biological relevance from earlier methods. We verify our approach on the well-characterized central metabolic network of *Saccharomyces cerevisiae*.

**Conclusion:**

We illustrate how **BOSS **offers insight into the functional organization of biochemical networks, facilitating the interrogation of cellular design principles and development of cellular engineering applications. Furthermore, we describe how growth is the best-fit objective function for the yeast metabolic network given experimentally-measured fluxes.

## Background

Systems-based approaches coupled with experimental data have facilitated greater understanding of large-scale biological systems [1,2]. For example, optimization procedures have recently been used to characterize systemic properties in biology, including phenotypic properties like growth rates and effects of gene knockouts [3-7]. One quantitative measure of a biological phenotype is the set of fluxes through all reactions within a biochemical network [8]. Specifically, flux balance analysis (FBA) is a constraints-based approach that calculates steady-state flux distributions [9-11].

FBA has traditionally been based on the premise that prokaryotes such as *Escherichia coli *have maximized their growth performance as a response to selective pressure [12]. Consequently, a common objective function in FBA of metabolic networks is the maximization of the rate of synthesis of biomass, a unit of measurement of cellular growth. However, as other types of networks and higher-order systems are interrogated, other objectives may be more accurate in predicting phenotypes. For example, other objective functions that have been previously considered in FBA include optimization of energy production or consumption [13] and byproduct synthesis [14]. By inferring objective functions of biological systems, cellular design principles may be studied and systems may be exploited for engineering of metabolic byproducts of commercial or medical value [15-20].

*In silico *frameworks for determining a most-likely objective function have previously been proposed. One such tool, named **ObjFind**, attempts to identify weightings, termed coefficients of importance (CoIs), on reaction fluxes within a network while minimizing the difference between the resultant flux distribution and known experimental fluxes [3]. In the **ObjFind **framework, a high CoI indicates a reaction that is more likely a component of the cellular objective function, given available experimental fluxes. However, **ObjFind **is unable to *a priori *define objectives, since in FBA the objective function is defined as a single reaction within the system (and represented within the stoichiometric matrix) and not a weighting on multiple reactions (i.e., a set of CoIs). For example, if the true objective reaction has not been experimentally characterized and is not included within the network reconstruction, **ObjFind **is unable to assign the highest CoI to it and instead chooses an alternate (and consequently suboptimal) reaction or set of reactions as constituting the objective function. Two recent efforts have further attempted to identify the most probable objective of a metabolic system from a set of possible objectives, in one case via a Bayesian-based probability ranking [21] and in the other case using an Euclidean metric [20]. However, like **ObjFind**, each of these methods requires that the stoichiometric network reconstruction include the true objective function as an existing reaction in order to yield meaningful predictions.

We present a novel framework, **Biological Objective Solution Search **(**BOSS**), for identifying objective functions of biological systems based on the stoichiometry of the underlying biochemical network(s) and known experimental flux data. In this framework, the biological objective function is a *de novo *reaction (column) that is added to the matrix **S **representing the stoichiometry of the underlying system. Subsequently, the flux through this particular objective reaction is optimized (maximized) as the objective function using the standard FBA approach. Notably, the objective reaction is not confined to be one of a subset of existing reactions, but rather is allowed to take on any form (e.g., an existing reaction, a combination of existing reactions, or a previously uncharacterized reaction) as specified by an optimization procedure. It is, however, confined to be *one *linear stoichiometric reaction whose coefficients are determined by the framework.

**Figure 1:**
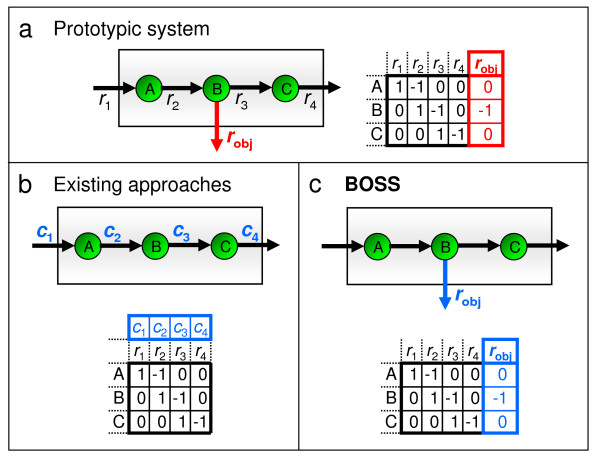
**The novelty of the BOSS optimization framework**. Panel (a) depicts a simple system comprised of three components and five reactions, including an objective reaction *r*_obj _denoted in red. Assuming a stoichiometric network reconstruction of this system (i.e., based on available literature) includes all three components and four of five reactions, excluding the objective reaction, panels (b) and (c) illustrate the objective functions that may be inferred by **ObjFind **and **BOSS**, respectively. Specifically, panel (b) shows how **ObjFind **generates a set of weightings, termed coefficients of importance (CoIs), on the reactions within the network. In this case, with the actual objective not in the stoichiometric reconstruction, **ObjFind **fails to assign a CoI to *r*_obj_. By contrast, panel (c) shows how **BOSS **generates a *de novo *stoichiometric reaction and recapitulates the actual system objective regardless of whether the actual objective is included as part of the stoichiometric reconstruction.

To illustrate this novelty of **BOSS **over existing methods (including the three described above), consider the simple system drawn in Figure 1(a). This system is comprised of three components and five reactions. The objective reaction is denoted as *r*_obj_. Assume that a network reconstruction based on available literature includes all three components and four of the five reactions but excludes the actual system objective, *r*_obj_, governing the resultant flux distribution. As shown in Figure 1(b), analyzing the network with an approach like **ObjFind **generates a rank-ordered list of the reactions that best describe the objective function. However, since *r*_obj _is unknown, the calculated objective will be some combination of weightings on *r*_1 _through *r*_4_, and the approach will fundamentally fail to capture the actual objective of the system *r*_obj_. However, as shown in Figure 1(c), by generating a *de novo *stoichiometric reaction as its objective function, **BOSS **would in theory be able to recapitulate the actual system objective.

We applied **BOSS **to the previously reconstructed *S. cerevisiae *central metabolic network [22,23] (see Additional file 1 for details of the network) to evaluate which objective reaction it infers for that system given a set of isotopomer flux data [23,24]. We compared the **BOSS**-derived objective reaction to the hypothesized system objective of precursor biomass synthesis. We considered two cases, one in which the hypothesized objective reaction (i.e., precursor biomass synthesis) was excluded from the system and another in which it was included within the set of known stoichiometric reactions. We further assessed how **BOSS **handles noise in experimental flux distributions. Finally, we compared our results to a negative control in which flux distributions comprised of randomly generated values were inputted into **BOSS**. Ultimately, we illustrate how **BOSS **extends existing objective function identification tools by inferring the objective reaction of a biochemical network *de novo *from internal state measurements, and how this tool therefore facilitates future study of cellular design principles and cellular engineering approaches.

## Methods

The framework that we present for identifying a putative objective function for a given biological system constitutes a single-level optimization problem. Specifically, **BOSS **integrates network stoichiometry, physico-chemical constraints such as reaction bounds, and experimental flux data to generate a novel stoichiometric reaction corresponding to the most likely objective function of the system. The framework and implementation strategies are described here.

### Flux Balance Analysis (FBA)

A key feature in the application of FBA is the optimization of an objective function subject to fundamental constraints on cellular function [9,11,25]. FBA requires a stoichiometric network reconstruction, usually represented as a stoichiometric matrix, **S**, in which components are delineated as rows, component interactions as columns, and stoichiometric relationships as coefficients (see Figure 1 for examples of biochemical networks and the corresponding **S **matrices). This reconstruction, coupled with reaction fluxes, comprises the principal constraint in FBA, i.e., at steady-state, for each component, the sum of the stoichiometric coefficients multiplied by the corresponding reaction fluxes must equal zero to ensure that mass is balanced within the system.

Commonly used objective functions (i.e., stoichiometric objectives or "objective reactions") in FBA of metabolic networks include biomass production [26,27], energy production or consumption [13], and byproduct production [14]. The objective function used in FBA is assumed to be linear by a first-order approximation, since this form simplifies computation and reduces the number of parameters to be defined in the objective experimentally. Changes in a linear objective under varying growth conditions have been observed [12].

FBA-predicted flux distributions have displayed agreement with experimentally-measured flux data in some cases [12]. Furthermore, FBA has yielded insights into optimal targets for metabolic engineering [15] and adaptive evolution of *E. coli *strains [28], among other applications [29]. Additionally, FBA has successfully predicted metabolic phenotypes of cellular systems by hypothesizing biomass production as the objective function [7,9,10,12,19,27,30-36]. However, other objectives may be equally successful at predicting phenotypes for particular conditions [20]. Furthermore, a metabolic objective may change at different stages of an organism's life cycle [37]. Some studies suggest a greater role for environmental factors in determining cellular objectives than is assumed when FBA is employed with a biomass production objective [38]. Ultimately, as additional network reconstructions are completed and experimental flux data are obtained, there is increasing interest in determining realistic objective functions to explain systemic behavior [18-20,39].

### Formulation of the Objective Function-Finding Algorithm BOSS

**Figure 2:**
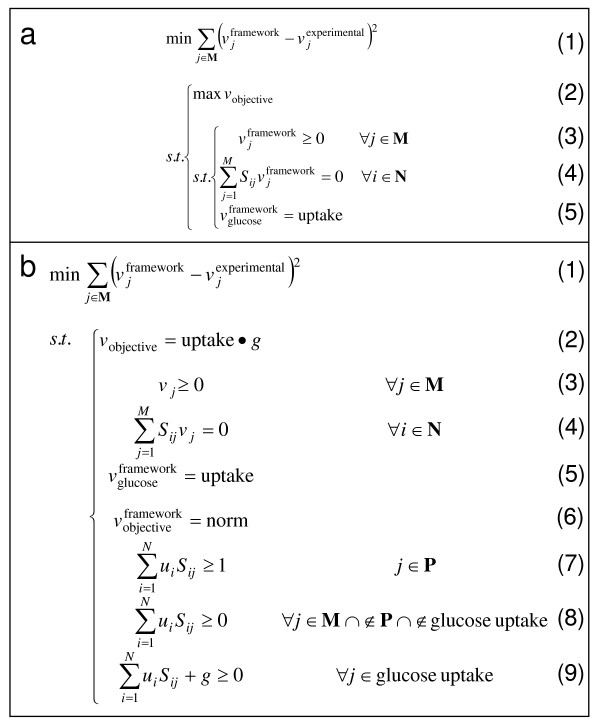
**The novel optimization framework implemented by BOSS**. Panel (a) illustrates the bi-level optimization problem that forms the basis for **BOSS**. This problem involves minimizing the sum-squared error between experimentally-measured (*in vivo*) and framework-computed (*in silico*) fluxes (line 1) subject to the fundamental flux balance analysis (FBA) problem (lines 2–5), i.e., the putative objective reaction is maximized (line 2) subject to physico-chemical (line 3) and other constraints (lines 4 and 5). In this framework: (1) **N **corresponds to the set of metabolites; (2) **M **to the set of reactions; (3) **P **to the set of putative objective reactions, usually a new column inserted into the stoichiometric matrix **S**, *S*_**i**, *j*_, with flux *v*_*j *_where *j *∈ **P**; (4) **v**^framework ^to the set of framework-computed fluxes; and (5) **v**^experimental ^to the set of experimentally-measured fluxes. Additionally, to normalize the flux data, the "input flux" corresponding to the uptake of the carbon source (e.g., glucose) $v_{\text{glucose}}^{\text{framework}}$ is set to a predetermined value called "uptake." Panel (b) illustrates the optimization problem in panel (a) reformulated as a single-level optimization problem via the duality theorem of linear programming (LP) [3, 40]. This novel framework for predicting objectives of biological systems is comprised of an objective that aims to minimize the sum-squared error between experimentally-measured and framework-computed fluxes (line 1) subject to a set of primal (lines 3 through 5) and dual constraints (lines 7 through 9), as well as two new constraints, one that sets the value of the primal and dual problems equivalent to one another (line 2) and another that normalizes the flux distribution by setting the flux corresponding to the new objective reaction to a specific value (line 6). The notations in panel (a) apply here as well. The decision variables in this optimization are: (1) the stoichiometric coefficients of the objective reaction, *S*_*i*, *j *_where *i *∈ **N **and *j *∈ **P**; (2) the framework-computed fluxes **v**^experimental^; (3) the dual variable *g *associated with the uptake constraint; and (4) the dual variables **u **indicating shadow prices on the mass balance constraints for each metabolite in the system.

The **BOSS **framework initially takes the form of a bi-level optimization problem that minimizes the sum-squared error between experimentally-measured (*in vivo*) fluxes and framework-computed (*in silico*) fluxes ("outer problem") (see Figure 2(a), line 1), subject to the condition that a cellular objective is simultaneously maximized ("inner problem") (lines 2–5). The inner problem takes the canonical form of a FBA problem, wherein an objective reaction introduced to the network is maximized (line 2) subject to thermodynamic (line 3), mass balance (line 4), and uptake (line 5) constraints. The coefficients of the objective reaction are unknown and part of the solution space.

In order to make this bi-level optimization problem computationally tractable, we reformulated it as a single-level optimization problem via the duality theorem of LP, as previously described [3,40] (see Figure 2(b)). Specifically, the revised form is comprised of an objective that aims to minimize the sum-squared error between experimentally-measured and framework-computed fluxes (line 1) subject to a set of primal (lines 3 through 5) and dual constraints (lines 7 through 9). The objective in line 1 is equivalent to the outer objective of the bi-level optimization problem (see Figure 2(a), line 1), and the primal constraints in lines 3 through 5 are equivalent to the constraints in the original bi-level optimization problem (see Figure 2(a), lines 3 through 5). Two additional constraints are included in the single-level optimization framework. Specifically, the value of the primal and dual problems are set equivalent to one another (line 2), and the flux distribution corresponding to the new objective reaction is normalized to a specific value (line 6). As described in the caption for Figure 2, the decision variables in this optimization are: (1) the stoichiometric coefficients of the objective reaction, *S*_*i*, objective _where *i *∈ **N**, or the set of metabolites, and "objective" denotes the new objective reaction; (2) the framework-computed fluxes **v**^experimental^; (3) the dual variable *g *associated with the uptake constraint; and (4) the dual variables **u **indicating shadow prices on the mass balance constraints for each metabolite in the system.

**Figure 3:**
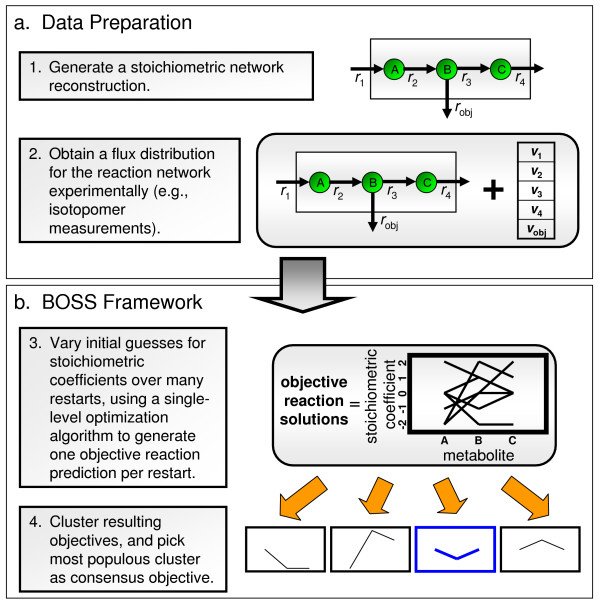
**The process flow for evaluating BOSS**. A four-step process for evaluating **BOSS **is illustrated. Specifically, in panel (a), we show how we (1) generate a stoichiometric matrix reconstruction for a biological system and (2) obtain experimental flux measurements for the reactions within the system. Note that when we tested **BOSS **on simulated noisy flux data in yeast central metabolism, each experimental flux was varied randomly via a normal distribution, with mean and standard deviation equivalent to the mean and standard deviation of the actual experimental fluxes. In panel (b), we show how (3) the reconstruction and experimental flux data are inputted into **BOSS **and stoichiometric coefficients for the objective reaction are identified through a multiple-restart strategy that utilizes random initial guesses for the coefficients. Additionally, (4) the resultant objectives with a low sum-squared error between the framework-computed flux data and the experimentally-measured flux data are clustered, and the most populous cluster is chosen as representative of the objective function of the system.

Due to the large number of decision variables, large solution space, existence of multiple local optima, and inherent non-convexity of the problem, we instituted a multiple restart approach, wherein the optimization is run multiple times, each time with different randomly-selected starting values for the decision variables (within a specified range) (see Figure 3(b), item 3). The output of this approach is a series of putative objective reactions, one for each restart. Objective reactions that yield a poor value in the outer optimization (i.e., for which the sum-squared error between experimental and calculated fluxes is relatively high) are removed from the solution set. The remaining putative objective reactions are then clustered into groups based on similarity, and the most populous cluster is chosen as the consensus objective reaction (see Figure 3(b), item 4, as well as Additional file 2). This last step seeks to eliminate solutions to the outer problem that constitute suboptimal local minima. It is assumed that the global minimum to the outer problem (i.e., the best match between **BOSS**-derived and experimental fluxes, thereby leading to the likely objective reaction) will draw from a larger portion of the initial state space than any local minimum, and thus will gather the greatest proportion of the restarts when solutions are clustered.

This single-level optimization with multiple restarts, followed by clustering and subsequent averaging of the most populous cluster, ultimately yields a stoichiometrically-weighted reaction for which the metabolic network is optimized. This reaction represents a best hypothesis for the network objective function based on available data about the system. Determination of the objective reaction in this manner offers a means to gain insight about network and sub-network objectives and behavior.

### Biological System Evaluated: The *S. cerevisiae *Central Metabolic Network

The previously reconstructed *S. cerevisiae *central metabolic network [22,23] was used to assess the **BOSS **framework (see Additional file 2). This network is a subset of the genome-scale *S. cerevisiae *metabolic reconstruction [41,42] and comprises the major carbohydrate metabolism pathways of glycolysis, pentose phosphate, and the citrate cycle, the principal energy metabolism pathway of oxidative phosphorylation, and a precursor biomass synthesis reaction. The network is comprised of 60 metabolites participating in 62 reactions, including six exchange reactions, 55 intracellular reactions, and one precursor biomass synthesis reaction.

A flux distribution for the central metabolic network was previously characterized experimentally [22,23]. This distribution was obtained by GC-MS tracing of ^13^C-labeled isotopomers, and corresponds to yeast cells cultivated in batch culture with glucose as the limiting substrate and at a maximum specific growth rate, *μ*_max_, of 0.37 h^-1^. (See [23] for complete experimental details.) The **BOSS **framework was applied to this data set.

In addition, the precursor biomass synthesis reaction, which balances the major metabolites from central metabolism that contribute to biomass, was hypothesized to be the "true" objective function of the system for testing the **BOSS **framework. This hypothesis was based on the underlying premise described previously that organisms have maximized their growth performance through natural selection [12]. The precursor biomass synthesis reaction was previously characterized experimentally [22,23]. It is important to note that **BOSS **does not require an assumption of an objective function, and we demonstrate below the success of **BOSS **at generating a hypothesized objective function *de novo*.

### Implementation Details

The general implementation scheme for **BOSS **is illustrated in Figure 3. Specifically, we inputted the stoichiometric network reconstruction and isotopomer flux data for the *S. cerevisiae *central metabolic network into **BOSS **(see Figure 3(a)) and assessed the objective reaction that it derived (see Figure 3(b)). We evaluated two conditions, one in which the hypothesized objective function of precursor biomass synthesis was included in the network reconstruction inputted into **BOSS **and another in which it was excluded. In the second case, **BOSS **yielded a zero-vector for the objective reaction, suggesting that the true objective was already a part of the network reconstruction. Therefore, we altered **BOSS **slightly to identify which of the reactions was the likely objective: we removed, one by one, each reaction flux from the set of experimental flux data (**v**^experimental ^in Figure 2), and identified in each case the sum-squared error between the **BOSS**-derived objective reaction and the reaction corresponding to the removed flux. The reaction that exhibited the smallest sum-squared error through this method was identified as the objective function. This approach was utilized to ensure that, if the actual system objective is already in the stoichiometric network reconstruction, the outer optimization problem of **BOSS **does not force flux to go through that reaction and rather allows the flux corresponding to the new objective function column to be maximized as part of **BOSS**.

We considered one additional *in silico *experiment to evaluate how well our framework handles the kind of variability that is characteristic of actual flux measurements. Specifically, we assessed how a varying amount of noise introduced to the set of experimental flux data affects the objective function derived by **BOSS**. (See Additional file 3 as well for a discussion of how **BOSS **handles a paucity of flux data, as applied to a prototypic system.) To perform this test, additional steps were performed during data preparation (see Figure 3(a), item 2). First, each experimental flux for the *S. cerevisiae *metabolic network was varied randomly via a normal distribution, with standard deviation equal to a set percent of the initial value of the flux. Different percent variances, ranging from five percent to 25 percent in increments of five percent, were considered. Fluxes with an initial value of zero were varied with a standard deviation equal to that of the smallest nonzero flux in the system. **BOSS **was fed these varied fluxes as "experimental" fluxes. To avoid biasing results toward one particular variant of the "experimental" fluxes, we repeated multiple times this process of randomly varying the experimental flux distribution. In other words, for any given percent variance, we tested multiple noisy flux data sets (n = 20). Consequently, when considering the results of any **BOSS **run on noisy flux data, we evaluated the coefficients of the objective reaction for each noisy flux data set independently.

#### Assessing the Results of BOSS

To assess the accuracy and efficacy of our **BOSS**-derived results, we considered two statistical measures. First, we computed the sum-squared error between the coefficients of the **BOSS**-derived objective reaction and the coefficients of the hypothesized objective reaction (in this case, precursor biomass synthesis), normalized to the magnitude of the hypothesized objective reaction column vector. This term is abbreviated SSE_**S **_throughout the manuscript. Second, we computed the sum-squared error between the reaction fluxes outputted by **BOSS **and the experimental fluxes inputted to the framework and specified as constraints. This term, which is equivalent to the function being minimized in the **BOSS **outer optimization problem, is abbreviated SSE_**v **_throughout the manuscript. Ideally, smaller values of SSE_**v **_should correspond to smaller values of SSE_**S**_, as smaller values of SSE_**S **_imply more accurate objective reactions.

#### Technical Implementation

Simulations of **BOSS **were run with GAMS v.22.5 on Microsoft Windows XP Professional and Linux (Centos5) machines. The MINOS5 NLP (non-linear programming) solver was utilized. Results were analyzed using code written in MATLAB v.7.5 (part of the MathWorks R2007b release package), with clustering functionality provided by the MATLAB Statistics Toolbox (as detailed in Additional file 2).

## Results and Discussion

The *S. cerevisiae *central metabolic network was used as an experimental system to evaluate the ability of **BOSS **to identify objective functions of biological systems. Additionally, the network was used to evaluate how well the framework handles noise in flux distributions, which is a characteristic of actual experimental measurements. These results were contrasted with randomly-generated flux data for the *S. cerevisiae *central metabolic network as a further validation of the **BOSS **framework. We summarize the results here.

### Validation of Objective Function Identification

**Figure 4:**
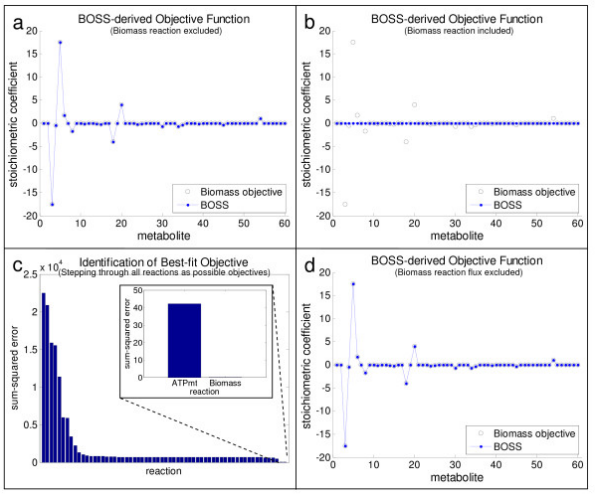
**Validating BOSS on the *S. cerevisiae *central metabolic network**. The results of **BOSS **(blue line and dots) when applied to the *S. cerevisiae *central metabolic network are illustrated and contrasted by the hypothesized objective reaction (i.e., precursor biomass synthesis) (open black circles). Panel (a) illustrates the results when the hypothesized objective reaction of precursor biomass synthesis is excluded from the network inputted into **BOSS**. Panel (b) depicts the results when the network is inputted into **BOSS **with the hypothesized objective reaction. Note that the entire experimental flux distribution was provided as input to **BOSS **in both cases. When the precursor biomass synthesis objective reaction was excluded from the network inputted into **BOSS**, the sum-squared error between the **BOSS**-derived objective reaction and the expected precursor biomass synthesis objective reaction, normalized to the magnitude of the precursor biomass synthesis objective reaction (SSE_**S**_), was 8.242 × 10^-29 ^(panel (a)). By contrast, when the precursor biomass synthesis objective reaction was included in the set of stoichiometric reactions inputted into **BOSS**, the SSE_**S **_for the **BOSS **solution was approximately 655.0 (panel (b)). Panel (c) depicts a plot of the sum-squared error between the **BOSS**-derived and the corresponding reaction when the flux for each of the 62 reactions in the system was excluded from the pool of experimental fluxes one by one. The smallest SSE_**S **_values were 42.20 and 4.210 × 10^-4 ^and corresponded to the ATP maintenance and biomass production reactions, respectively. Consequently, as shown in panel (d), **BOSS **was able to recapitulate the hypothesized objective reaction of precursor biomass synthesis with a SSE_**S **_= 4.210 × 10^-4 ^when the reaction was included in the network stoichiometry but its experimental flux was removed from the available flux data.

In inferring the objective function for the *S. cerevisiae *metabolic network with **BOSS**, two conditions were evaluated. In the first one, the hypothesized objective reaction (i.e., precursor biomass synthesis) was excluded from the stoichiometric network reconstruction. **BOSS **identified coefficients approximately equal to that of the precursor biomass synthesis reaction (see Figure 4(a)). The sum-squared error between the **BOSS**-computed objective reaction and the experimentally-derived precursor biomass synthesis reaction, normalized to the magnitude of the precursor biomass synthesis reaction (SSE_**S**_), was 8.242 × 10^-29^. The magnitude of this SSE_**S **_suggests that **BOSS **derives an objective reaction equivalent to the precursor biomass synthesis reaction, well within the limits of numerical tolerance.

We also implemented **BOSS **on the complete *S. cerevisiae *central metabolic network, i.e., without removing the precursor biomass synthesis reaction. When the precursor biomass synthesis reaction was thus included in the set of stoichiometric reactions, **BOSS **yielded a zero-vector for the objective reaction (SSE_**S **_= 655.0), suggesting that the actual system objective was already a part of the stoichiometric network reconstruction (see Figure 4(b)). Subsequently, we individually removed each of the reaction fluxes from the pool of experimental fluxes defined in the outer optimization problem of **BOSS **(i.e., **v**^experimental ^in Line 1 of Figure 2(a)) and observed the resultant stoichiometric coefficients that **BOSS **derived for the objective function. SSE_**S **_values between the reaction whose flux was removed from **v**^experimental ^and the **BOSS**-derived objective reaction given removal of that flux were compared for each reaction in the system (see Figure 4(c)). The reaction with the smallest SSE_**S **_(between the **BOSS**-derived objective and the experimentally-characterized reaction whose flux was excluded) was the hypothesized system objective of precursor biomass synthesis (SSE_**S **_= 4.210 × 10^-4^). This result further validated the ability of **BOSS **to identify objective reactions with previously known as well as unknown stoichiometry (see Figures 4(c) and 4(d)). Interestingly, the reaction with the next smallest SSE_**S **_(= 42.20) was the ATP maintenance reaction, a reaction that has also been evaluated as an objective of metabolic networks under some conditions [3,21,43]. All other reactions in the network had SSE_**S **_values that were at least an order of magnitude higher. This key result is also a direct validation of the hypothesis that biomass production is a reasonable objective function for many metabolic networks; with the experimentally-generated flux data, **BOSS **derived an objective function highly correlated with biomass production although there was no such assumption *a priori*.

It is important to note that application of FBA to the *S. cerevisiae *metabolic network does *not *yield the experimental flux distribution measured by [23]. Because FBA problems have large convex solution spaces, many different flux distributions yield equally optimal results. Therefore, it is significant that **BOSS **is able to infer the correct coefficients for the precursor biomass synthesis reaction in spite of this difference. Furthermore, although maximization of biomass synthesis is hypothesized as the objective of a metabolic network, the experimental flux data are calculated based on isotopomer measurements and the underlying system stoichiometry but is not biased toward this objective. Thus, the characterization of biomass as the system objective by **BOSS **is a novel validation of this theoretical assumption.

### Accounting for Limitations in Experimental Measurements: Noisy Flux Data

To account for current limitations in experimental (isotopomer) technologies for measuring reaction fluxes, the *S. cerevisiae *central metabolic network was evaluated by **BOSS **when different levels of noise were introduced into the experimental flux data. Different percentages of deviation for the flux data were evaluated to determine how quickly the accuracy of the **BOSS**-computed objective reaction degrades with noisy data.

**Figure 5:**
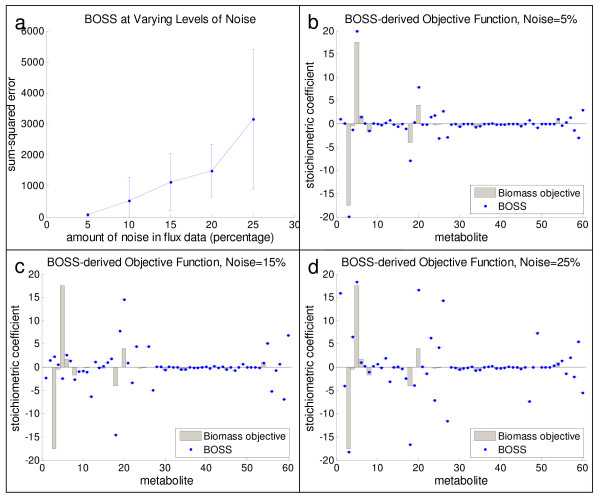
**Evaluating noise in the flux data in the *S. cerevisiae *central metabolic network for the precursor biomass synthesis objective reaction**. The results of **BOSS **when noisy flux data are supplied as input to the framework are illustrated in the context of the precursor biomass synthesis objective reaction. Panel (a) illustrates the sum-squared error between the computed objective reaction and the hypothesized objective reaction of precursor biomass synthesis, when normalized to the magnitude of the precursor biomass synthesis reaction. The mean and standard deviation represented by the error bars for SSE_**S **_at each of the percent variances are based on the 20 independent runs at each level of noise (i.e., 20 independent flux distributions with equivalent amounts of noise randomly inserted). Panels (b), (c), and (d) illustrate the hypothesized objective reaction (gray bars) and the **BOSS**-derived objective reaction (blue dots/lines) when the reaction fluxes in the network are varied by 5, 15, and 25 percent of their actual values, respectively. As the variance in the fluxes increases, the performance of **BOSS **degrades. Note that, as described previously in the context of Figures 4(c) and 4(d), to avoid biasing results, the hypothesized objective reaction of precursor biomass synthesis was included in the network inputted into **BOSS**, and the pool of experimental fluxes consisted of all fluxes with the exception of the precursor biomass synthesis reaction flux.

The results for the *S. cerevisiae *network when varying levels of noise are introduced into the individual flux values are illustrated in Figure 5. For the purposes of this evaluation, based on the results described above, the hypothesized objective reaction of precursor biomass synthesis was included as part of the underlying network stoichiometry inputted into **BOSS**, and the set of experimental fluxes consisted of all fluxes except for the flux corresponding to this reaction. Figure 5(a) illustrates the normalized sum-squared error between the **BOSS**-derived objective reaction and the hypothesized objective function of precursor biomass synthesis (SSE_**S**_) for different percent variances in experimental flux data, ranging from five percent to 25 percent in increments of five percent. (As described above, for each increment, 20 unique "initial" flux distributions, each with the same level of "noise," were computed, and the results for each of these 20 flux distributions were treated independently of the others. Therefore, the plot in Figure 5(a) illustrates the mean SSE_**S **_across these 20 distributions, as well as the associated standard deviation within SSE_S _across these 20 distributions.) Up to about 15 percent variance in the flux data, SSE_**S **_< 1000, i.e., **BOSS **is able to generate reconstructions of the hypothesized objective reaction orders of magnitude more accurately than when random flux data are inputted into **BOSS **(see "Control Case: Random Flux Data" below). As expected, with increasing noise, the results of **BOSS **exhibit increasing mean SSE_**S **_values and the variability of SSE_**S **_across different initial flux sets is higher. These data support the utility of our framework in identifying objective functions for biological systems given the current state of experimental flux measurements.

To further highlight **BOSS**'s performance on noisy flux data, snapshots of the objective reactions that it generated at different flux variances are illustrated. Specifically, Figures 5(b), 5(c), and 5(d) correspond to the coefficients identified by **BOSS **(blue error bars) overlaid on the precursor biomass synthesis reaction (gray bars) for flux variances of 5, 15, and 25 percent, respectively. (Note that, for each flux variance, 20 unique "initial" flux distributions were computed. Consequently, the values for the coefficients derived by **BOSS **correspond to a representative result for one of these 20 initial flux sets.) For smaller flux variances, the pattern of the coefficients identified by **BOSS **follows that of the hypothesized network objective of precursor biomass synthesis that was used to generate the noisy flux data (see Figure 5(b)). As expected, the more noisy the flux data, the less precise the objective reaction identified by the framework (see Figure 5(c)).

It is important to note that this analysis involves artificially adding noise to an experimental flux distribution. Although the original experimental fluxes are based on a steady-state metabolic model of yeast central metabolism, it is likely that there is some degree of noise already within the data set as part of the experimental protocol. Consequently, the studies with noise presented here include even higher degrees of noise than the five to 25 percent spectrum evaluated. Nevertheless, the results suggest that **BOSS **performs well, to an extent, with noisy experimental data.

#### Control Case: Random Flux Data

To place these results in context, randomly-generated flux values coupled with the underlying network stoichiometry for the *S. cerevisiae *central metabolic network were inputted into the **BOSS **framework as a negative control. These fluxes were generated by specifying a normal distribution statistically similar to the characteristics of the experimental flux distribution. Specifically, the randomly-generated fluxes were centered at a mean of 6.697 mmol/g·h with a standard deviation of 9.521 mmol/g·h, identical to the mean and standard deviation of the experimental fluxes reported in [23,24]. For this test of random flux data, no reaction was removed from the stoichiometric network reconstruction inputted into **BOSS **to avoid biasing results in any way toward a particular objective. However, to ensure that flux was driven through the objective reaction, thus minimizing SSE_**v **_(see discussion above pertaining to Figures 4(b), 4(c), and 4(d)), the flux for the precursor biomass synthesis objective reaction was not included in the pool of experimental flux data inputted into **BOSS**, as described earlier. A total of 100 randomly-generated flux distributions, each with mean and standard deviation consistent with the experimental flux data, were evaluated, and the SSE_**S **_between the hypothesized objective reaction of precursor biomass synthesis and the **BOSS**-derived objective reaction was 2.076 × 10^4^. In addition, the SSE_**v **_was 962.9. These results are orders of magnitude higher than when actual experimental flux data, or even noisy experimental data, are specified (see "Validation of Objective Function Identification" above), thus further confirming the utility of **BOSS **in inferring objective functions of biological systems with internal state measurements.

## Conclusion

We present a novel framework called **Biological Objective Solution Search **(**BOSS**) that integrates network stoichiometry and experimental flux data to determine the most likely objective function for a given biological system. We illustrate the utility of **BOSS **on a model of *S. cerevisiae *central metabolism with a variety of input conditions, including experimental (isotopomer) flux data, varied experimental flux data meant to mimic noise in experimental measurements, and randomly-generated fluxes. When the underlying network stoichiometry and all reaction fluxes are specified, **BOSS **identifies an objective reaction with a normalized sum-squared error between the computed objective and the hypothesized objective (i.e., precursor biomass synthesis) (SSE_**S**_) of 4.210 × 10^-4^. Additionally, when noise is introduced into the flux data to simulate the types of error observed in experimental flux measurements, **BOSS **identifies the objective reaction with a SSE_**S **_of less than 1000 for up to 15 percent experimental flux noise. Thus, we show how **BOSS **can integrate network stoichiometry and internal state measurements, including in the case of noisy data, to predict the objective function of a biological system.

Interestingly, researchers have theorized that, through evolution, metabolic systems have optimized for biomass production [12]. Here we illustrate that our framework derives this hypothesized objective reaction, precursor biomass synthesis, based on the underlying network stoichiometry and a set of experimentally-measured flux data. This observation validates the **BOSS **framework and it further confirms the precursor biomass synthesis reaction as an objective of yeast central metabolism.

Current challenges remain in the implementation of the **BOSS **algorithm. The transformation of a bi-level to a single-level optimization via the strong duality theorem (see "Methods") introduces a high degree of nonlinearity into the problem and non-convexity into the solution space, rendering the **BOSS **algorithm difficult for nonlinear solvers to tackle. Further, the structure of the inner and outer problems suggests that the solution space for a **BOSS **problem is not smooth, but rather likely contains discontinuous rifts in the value of the optimization parameter (SSE_**v**_) as one moves smoothly through the state space. These factors complicate the ability of **BOSS **to determine a global optimum with a small number of restarts, resulting in several hours to days of simulation time depending on the system and input conditions. Perhaps stronger nonlinear solvers, more powerful dedicated computers, and more run-time would yield better predictions of objective functions. These challenges will need to be addressed as **BOSS **is scaled up to genome-scale applications.

Another hurdle to the meaningful application of **BOSS **is the paucity of large-scale experimentally-derived flux sets in literature. Isotopomer studies, as well as ^13^C-constrained fluxomics studies, tend to use simplified models of central metabolism that lump reactions together and thus fail to define a distinct flux for each reaction in the system [44-47]. These simplifications have been necessary due to experimental and computational hurdles, but they limit the degree to which experimental flux data can be used by **BOSS**. As more powerful fluxomics techniques are developed, more complete flux distribution maps will become available for analysis by **BOSS **[48].

**BOSS **is distinguished from preexisting objective-searching algorithms in its ability to define an objective reaction absent previous knowledge of the stoichiometric structure of the objective function [3,21]. This attribute could prove useful in analyzing signaling and transcriptional regulatory networks, as their objectives are likely to be more loosely defined and poorly understood than those of metabolic networks [32,49]. Likewise, studying objectives of multiple metabolically-interacting species or compartmentalized metabolic processes (e.g., mitochondrial metabolism) offers fruitful challenges that cannot be described as simply biomass production. Such analyses could lead to a greater appreciation of the driving evolutionary forces that govern these cellular processes, and could help explain how the whole-cell objective originates. A framework that predicts objectives of a biological system based on known properties of the system (e.g., the stoichiometry of the system and associated reaction fluxes) thus has the potential to address many critical and timely questions in biology.

## Authors' contributions

EG and MO conceived of the study and participated in its design and implementation. JP conceived of the study and participated in its design and coordination. AB and CM helped with key analyses of the data. All authors read and approved of the final manuscript.

## Supplementary Material

Additional File 1 *Saccharomyces cerevisiae *metabolism. A description of the *S. cerevisiae *central metabolic network from [22], including reaction and metabolite listing, is provided. Click here for file

Additional File 2 Clustering in **BOSS**. The clustering component of the **BOSS **framework, as implemented in MATLAB, is described. Click here for file

Additional File 3 Evaluating data integration in **BOSS**. **BOSS **is applied to a prototypic system designed to illustrate the ability of the framework to infer an objective function by synthesizing information from multiple experimental conditions. Click here for file
