# Supplementary material for: Predicting biological system objectives de novo from internal state measurements
Source: BMC Bioinformatics. 2008 Jan 24;9:43. doi: 10.1186/1471-2105-9-43 (PMC2258290; doi:10.1186/1471-2105-9-43)
Supplement: Additional File 1 — Saccharomyces cerevisiae metabolism. A description of the S. cerevisiae central metabolic network from [22], including reaction and metabolite listing, is provided. [file 1471-2105-9-43-S1.pdf]

ADDITIONAL FILE 1: *SACCHAROMYCES CEREVISIAE* METABOLISM

Our framework was applied to the *Saccharomyces cerevisiae* central metabolic network [1, 2]. This network is a subset of the genome-scale *S. cerevisiae* metabolic reconstruction [3, 4] and comprises the major carbohydrate metabolism pathways of glycolysis, pentose phosphate, and the citrate cycle, the principal energy metabolism pathway of oxidative phosphorylation, and a precursor biomass synthesis reaction. The network is comprised of 60 metabolites (see Table S1.1) participating in 62 reactions (see Table S1.2), including six exchange reactions, 55 intracellular reactions, and one precursor biomass synthesis reaction.

A flux distribution previously obtained through GC-MS analysis of ^{13}C isotopomer tracing through the network [1, 2] was utilized for the **BOSS** analyses (see Table S1.2). Glucose input was constrained to a fixed uptake value (=15.9 mmol/gDW/hr), and a maximum specific growth rate, μ_{max}, of 0.37 h^{-1}, was assumed to match the conditions under which the flux distribution was obtained. See [1, 2] for additional details.

In addition, the precursor biomass synthesis reaction, which balances the major metabolites from central metabolism that contribute to biomass, was hypothesized to be the objective reaction of the system, based on the premise that organisms such as yeast have optimized their growth function through evolution [5]. This reaction was previously characterized experimentally [1, 2].

References

1. Wang L, Hatzimanikatis V: **Metabolic engineering under uncertainty--II: analysis of yeast metabolism**. *Metab Eng* 2006, **8**(2):142-159.
2. Gombert AK, Moreira dos Santos M, Christensen B, Nielsen J: **Network identification and flux quantification in the central metabolism of Saccharomyces cerevisiae under different conditions of glucose repression**. *J Bacteriol* 2001, **183**(4):1441-1451.
3. Herrgard MJ, Lee BS, Portnoy V, Palsson BO: **Integrated analysis of regulatory and metabolic networks reveals novel regulatory mechanisms in Saccharomyces cerevisiae**. *Genome Res* 2006, **16**(5):627-635.
4. Duarte NC, Herrgard MJ, Palsson BO: **Reconstruction and validation of Saccharomyces cerevisiae iND750, a fully compartmentalized genome-scale metabolic model**. *Genome Res* 2004, **14**(7):1298-1309.

5. Segre D, Vitkup D, Church GM: **Analysis of optimality in natural and perturbed metabolic networks**. *Proc Natl Acad Sci U S A* 2002, **99**(23):15112-15117.

Figure captions

Table S1.1. Metabolites in the *S. cerevisiae* central metabolic network. A listing of the 60 metabolites in the *S. cerevisiae* central metabolic network derived from [1, 2] is shown.

Table S1.2. Reactions in the *S. cerevisiae* central metabolic network. A listing of the 62 reactions in the *S. cerevisiae* central metabolic network derived from [1, 2] is shown. Additionally, reaction fluxes that were specified as input to **BOSS**, corresponding to isotopomer flux data from [1, 2], are listed.

Table S1.1

No.	Abbreviation	Full name	Location
1	13PDG(c)	biphosphoglycerate	Cytosol
2	2PG(c)	2-phosphoglycerate	Cytosol
3	3PG(c)	3-phosphoglycerate	Cytosol
4	AC(c)	acetate	Cytosol
5	ACAL(c)	acetaldehyde	Cytosol
6	ACAR(c)	acetylcarnitine	Cytosol
7	ACAR(m)	acetylcarnitine	Mitochondria
8	ACCOA(c)	acetyl-CoA	Cytosol
9	ACCOA(m)	acetyl-CoA	Mitochondria
10	ADP(c)	ADP	Cytosol
11	ADP(m)	ADP	Mitochondria
12	AKG(m)	2-oxoglutarate	Mitochondria
13	AMP(c)	AMP	Cytosol
14	ATP(c)	ATP	Cytosol
15	ATP(m)	ATP	Mitochondria
16	Biomass	biomass	Extracellular
17	CAR(c)	carnitine	Cytosol
18	CAR(m)	carnitine	Mitochondria
19	CIT(m)	citrate	Mitochondria
20	COA(c)	coenzyme A	Cytosol
21	COA(m)	coenzyme A	Mitochondria
22	D6PGL(c)	glucono-1,5-lactone 6-phosphate	Cytosol
23	E4P(c)	erythrose 4-phosphate	Cytosol
24	ETH(c)	ethanol	Cytosol
25	F6P(c)	fructose 6-phosphate	Cytosol
26	FAD(m)	FAD	Mitochondria
27	FADH2(m)	FADH2	Mitochondria
28	FDP(c)	fructose 1,6-diphosphate	Cytosol
29	FERI(mm)	2 ferricytochrome c	Mitochondrial membrane
30	FERO(mm)	2 ferrocytochrome c	Mitochondrial membrane
31	FUM(m)	fumarate	Mitochondria
32	G6P(c)	glucose 6-phosphate	Cytosol
33	GL(c)	glycerol	Cytosol
34	GLC(c)	glucose	Cytosol
35	GLC(ex)	glucose	Extracellular
36	H(c)	H^+	Cytosol
37	H(m)	H^+	Mitochondria
38	ICIT(m)	isocitrate	Mitochondria
39	MAL(m)	malate	Mitochondria
40	NAD(c)	NAD	Cytosol
41	NAD(m)	NAD	Mitochondria
42	NADH(c)	NADH	Cytosol
43	NADH(m)	NADH	Mitochondria
44	NADP(c)	NADP	Cytosol
45	NADPH(c)	NADPH	Cytosol
46	OA(c)	oxaloacetate	Cytosol
47	OA(m)	oxaloacetate	Mitochondria
48	PEP(c)	phosphoenolpyruvate	Cytosol
49	PYR(c)	pyruvate	Cytosol
50	PYR(m)	pyruvate	Mitochondria
51	Q(mm)	ubiquinone	Mitochondrial membrane
52	QH2(mm)	ubiquinol	Mitochondrial membrane
53	R5P(c)	ribose 5-phosphate	Cytosol
54	RL5P(c)	ribulose 5-phosphate	Cytosol
55	S7P(c)	sedoheptulose 7-phosphate	Cytosol
56	SUC(m)	succinate	Mitochondria
57	SUCCOA(m)	succinyl-CoA	Mitochondria
58	T3P1(c)	glyceraldehydes-3-phosphate	Cytosol
59	T3P2(c)	glycerone phosphate	Cytosol
60	X5P(c)	xylose 5-phosphate	Cytosol

Table S1.2

No.	Abbreviation	Full name	Reaction
1	HXT	hexose transporter	-1 GLC(ex) 1 GLC(c)
2	HXK	hexokinase	-1 GLC(c) -1 ATP(c) 1 G6P(c) 1 ADP(c)
3	ZWF	glucose-6-phosphate-1-dehydrogenase	-1 G6P(c) -1 NADP(c) 1 D6PGL(c) 1 NADPH(c)
4	GND	6-phosphogluconate dehydrogenase	-1 NADP(c) -1 D6PGL(c) 1 NADPH(c) 1 RL5P(c)
5	RPE	ribulose-5-phosphate	-1 RL5P(c) 1 X5P(c)
6	RKI	Ribose-5-phosphate isomerase	-1 RL5P(c) 1 R5P(c)
7	PGI	glucose-6-phosphate isomerase	-1 G6P(c) 1 F6P(c)
8	PFK	phosphofructokinase	-1 ATP(c) -1 F6P(c) 1 ADP(c) 1 FDP(c)
9	TKL2	transketolase	-1 X5P(c) -1 E4P(c) 1 F6P(c) 1 T3P1(c)
10	TAL	transaldolase	-1 T3P1(c) -1 S7P(c) 1 F6P(c) 1 E4P(c)
11	FBA	fructose-biphosphate aldolase	-1 FDP(c) 1 T3P1(c) 1 T3P2(c)
12	TPI	triose phosphate isomerase	-1 T3P2(c) 1 T3P1(c)
13	TKL1	transketolase	-1 X5P(c) -1 R5P(c) 1 T3P1(c) 1 S7P(c)
14	TDH	glyceraldehyde-3-phosphate dehydrogenase	-1 T3P1(c) -1 NAD(c) 1 13PDG(c) 1 NADH(c)
15	GPD	glycerol-3-phosphate dehydrogenase	-1 T3P2(c) -1 NADH(c) 1 NAD(c) 1 GL(c)
16	PGK	phosphoglycerate kinase	-1 13PDG(c) -1 ADP(c) 1 3PG(c) 1 ATP(c)
17	GPM	phosphoglycerate mutase	-1 3PG(c) 1 2PG(c)
18	ENO	enolase	-1 2PG(c) 1 PEP(c)
19	PYK	pyruvate kinase	-1 PEP(c) -1 ADP(c) 1 PYR(c) 1 ATP(c)
20	PDC	pyruvate decarboxylase	-1 PYR(c) 1 ACAL(c)
21	ADH	alcohol dehydrogenase	-1 ACAL(c) -1 NADH(c) 1 ETH(c) 1 NAD(c)
22	ALD	aldehyde dehydrogenase	-1 ACAL(c) -1 NAD(c) 1 AC(c) 1 NADH(c)
23	ACE	acetate transporter	-1 AC(c) 1 AC(ex)
24	ACS	acetyl-coA synthase	-1 AC(c) -1 COA(c) -1 ATP(c) 1 ACCOA(c) 1 AMP(c)
25	CAT	carnitine *o*-acetyltransferase	-1 ACCOA(c) -1 CAR(c) 1 ACAR(c) 1 COA(c)
26	PYR	pyruvate carrier	-1 PYR(c) 1 PYR(m)
27	PYC	pyruvate carboxylase	-1 PYR(c) -1 ATP(c) 1 ADP(c) 1 OA(c)
28	OA	oxaloacetate carrier	-1 OA(c) 1 OA(m)
29	PDA	pyruvate dehydrogenase	-1 PYR(m) -1 COA(m) -1 NAD(m) 1 ACCOA(m) 1 NADH(m)
30	YAT	carnitine *o*-acetyltransferase	-1 COA(m) -1 ACAR(m) 1 ACCOA(m) 1 CAR(m)
31	CAR	carnitine diffusion	-1 CAR(m) 1 CAR(c)
32	ACAR	acetylcarnitine diffusion	-1 ACAR(c) 1 ACAR(m)
33	CIT	citrate synthase	-1 ACCOA(m) -1 OA(m) 1 COA(m) 1 CIT(m)
34	ACO	aconitase	-1 CIT(m) 1 ICIT(m)
35	IDH	isocitrate dehydrogenase	-1 ICIT(m) -1 NAD(m) 1 AKG(m) 1 NADH(m)
36	KGD	α-ketoglutarate dehydrogenase	-1 AKG(m) -1 COA(m) -1 NAD(m) 1 SUCCOA(m) 1 NADH(m)
37	LSC	succinate-CoA ligase	-1 SUCCOA(m) -1 ADP(m) 1 COA(m) 1 SUC(m) 1 ATP(m)
38	SDH	succinate dehydrogenase	-1 SUC(m) -1 FAD(m) 1 FUM(m) 1 FADH2(m)
39	FUM	fumaratase	-1 FUM(m) 1 MAL(m)
40	MDH	malate dehydrogenase	-1 OA(m) -1 NADH(m) 1 MAL(m) 1 NAD(m)
41	MAE	malic enzyme	-1 MAL(m) -1 NAD(m) 1 PYR(m) 1 NADH(m)
42	ADK	adenylates kinase	-1 ATP(c) -1 AMP(c) 1 ADP(c)
43	ATPmt	ATP maintenance	-1 ATP(c) 1 ADP(c)
44	NDI	NADH dehydrogenase	-1 Q(mm) -1 NADH(m) 1 QH2(mm) 1 NAD(m)
45	NDH	external NADH dehydrogenase	-1 Q(mm) -1 NADH(c) 1 QH2(mm) 1 NAD(c)
46	FDH	FADH2 dehydrogenase	-1 Q(mm) -1 FADH2(m) 1 QH2(mm) 1 FAD(m)
47	QCR	ubiquinol cytochrome *C* reductase	-2 QH2(mm) -2 FERI(mm) -1.5 H(m) 2 Q(mm) 2 FERO(mm) 1.5 H(c)
48	NCP	NADPH reductase	-2 FERI(mm) -1 NADPH(c) 2 FERO(mm) 1 NADP(c)
49	COX	cytochrome *C* oxidase	-2 FERO(mm) -3 H(m) 2 FERI(mm) 3 H(c)
50	ASN	ATP synthase	-1 ADP(m) -3 H(c) 1 ATP(c) 3 H(m)
51	AAC	ADP/ATP carrier protein	-1 ADP(c) -1 ATP(m) -1 H(m) 1 ATP(c) 1 ADP(m) 1 H(c)
52	GLCex	glucose exchange	1 GLC(ex)
53	Biomass	precursor biomass reaction	-0.44 G6P(c) -0.15 R5P(c) -0.09 E4P(c) -0.26 T3P1(c) -0.19 PEP(c) -0.06 PYR(c) -0.65 ACCOA(c) -0.64 PYR(m) -0.37 OA(c) -0.13 ACCOA(m) -0.4 AKG(m) -17.53 ATP(c) -1.76 NADPH(c) -3.98 NAD(c) 1 Biomass 17.53 ADP(c) 1.76 NADPH(c) 3.98 NADH(c)
54	Biomassex	biomass exchange	-1 Biomass
55	GLcex	glycerol exchange	-1 GL(c)
56	ETHcex	ethanol exchange	-1 ETH(c)
57	CoAcex	coenzyme A (cytosol) exchange	1 CoA(c)
58	CoAmex	coenzyme A (mitochondria) exchange	1 CoA(m)
59	HmHct	H^+ exchange	-1 H(m) 1 H(c)
60	Oamex	oxaloacetate (mitochondria) exchange	1 OA(m)
61	FERIt	2 ferricytochrome c exchange	-1 FERO(mm) 1 FERI(mm)
62	NADHmt	NADH (mitochondria) exchange	-1 NADH(m) 1 NAD(m)
