# Supplementary material for: Predicting biological system objectives de novo from internal state measurements
Source: BMC Bioinformatics. 2008 Jan 24;9:43. doi: 10.1186/1471-2105-9-43 (PMC2258290; doi:10.1186/1471-2105-9-43)
Supplement: Additional File 2 — Clustering in BOSS. The clustering component of the BOSS framework, as implemented in MATLAB, is described. [file 1471-2105-9-43-S2.pdf]

ADDITIONAL FILE 2: CLUSTERING IN BOSS

Clustering in the **BOSS** framework was performed using the MATLAB "CLUSTER" function, found in the MATLAB Statistics Toolbox. This function, in conjunction with the functions "CORRDIST" and "CLUSTERTREE", perform hierarchical clustering given an input of a maximum number of clusters ("maxclust"). This "maxclust" input specifies for the CLUSTER algorithm the number of clusters into which the data are expected to group. In order to determine the "maxclust" setting for our data, we performed an initial analysis of the clustering outputs for several sets of **BOSS** results and ultimately determined that, within a certain range, the post-clustering results were relatively stable over a range of "maxclust" values.

A representative example is given, taken from the prototypic system (described in Additional file 3) with wild-type (abbreviated WT), reaction 7 knockout (v_7 knockout or KO-7), and reaction 9 knockout (v_9 knockout or KO-9) data taken as the "experimental" flux data set. This trial includes 1000 iterations of the optimization framework (i.e., **BOSS**), with different starting values for each restart (see the manuscript and Additional file 3 for details). The outputs from **BOSS**, prior to any clustering, are presented in Figure S2.1. In this case, it is clear by simple inspection of the histogram of SSE_S values (i.e., the sum-squared error between the **BOSS**-derived objective reaction and the hypothesized objective reaction vector, normalized to the magnitude of the hypothesized objective reaction vector) for all 1000 restarts that one solution dominates (see Figure S2.2). This result was apparent for most data sets, although the consensus solution did not always dominate as strongly.

To perform more detailed analysis, results were clustered with different "maxclust" values, and the resulting numbers of solutions in the most populous cluster were plotted. The sum, across all metabolites, of standard deviations per metabolite coefficient between all of the solutions grouped into the most populous (consensus) cluster, was also plotted (Figure S2.3). In this plot, the standard deviation metric (blue boxes) gives the relative tightness of the most populous cluster (i.e., a low standard deviation corresponds to a very tightly coupled cluster), and the metric "number of solutions in heftiest cluster" (red circles) gives the size of the most populous cluster. For "maxclust" values of 1 through 9, the numbers of solutions in the heftiest cluster

drops significantly with each increase in "maxclust" value (Figure S2.3, "significant drop phase"). This result is typical when the numbers of clusters is low, since a low cluster number forces dissimilar solutions to be clustered together, simply because there are not enough clusters to separate the data. As the cluster number expands, these dissimilar solutions quickly drop away from the cluster. At some point (here 10 clusters), the size of the most populous cluster abruptly stops changing significantly with changes in "maxclust," indicating that a stable point is attained (Figure S2.3, "leveled off phase"). As the cluster number is further increased, the number of solutions in the consensus cluster remains relatively stable. Since the "standard deviation in the most populous cluster" metric does not change significantly within this leveled-off phase, we assume that the solutions are similar, and that the most populous cluster only loses solutions because we are expanding the clustering size, which allows similar solutions to de-group despite their similarity.

We thus assume that the best solution lies in the leveled-off phase, preferably at fewer clusters, since this keeps the maximum number of similar solutions in the cluster. This detailed analysis was not performed for every trial, since it was clear from analyzing multiple data sets that as long as a "maxclust" was picked in the leveled-off phase, the solution cluster would be relatively insensitive to changes in the "maxclust" value. A "maxclust" value of 50 was picked for most trials of 500 to 1000 restarts with the prototypic system described in Additional file 3, while a "maxclust" value of 20 was chosen for most large runs with the *S. cerevisiae* central metabolic network described in the manuscript and Additional file 1.

Figure S2.4 presents the raw solutions that were selected in the consensus cluster for "maxclust" values of 5, 50, and 250 for the prototypic system described in Additional file 3 (Figures S2.4(a), S2.4(b), and S2.4(c), respectively). Clearly, a "maxclust" of 5 is too low, since a significant number of dissimilar results are clustered in this consensus cluster. The consensus cluster for a "maxclust" of 50 does not significantly differ in shape from that of a "maxclust" of 250, which reinforces the previous analysis (Figure S2.3). Figure S2.4(d) shows the averaged consensus cluster from a "maxclust" value of 50, which is what is also presented in the mansucript.

Figure captions

Figure S2.1. All solutions prior to clustering. The raw objective reactions computed by **BOSS** over many different restarts are shown for the prototypic system (see Additional file 3) when wild-type (WT), reaction 7 knockout (v_7 knockout or KO-7), and reaction 9 knockout (v_9 knockout or KO-9) data are supplied to the framework.

Figure S2.2. Normalized sum-squared error values. A histogram of the sum-squared error between the **BOSS**-derived objective reaction and the expected objective reaction, normalized to the magnitude of the expected objective reaction vector, is plotted for the various **BOSS** results prior to any clustering.

Figure S2.3. Evaluating different numbers of clusters. The number of solutions (red circles) and the standard deviation of the coefficients (blue circle) within the consensus cluster are plotted as a function of the number of clusters.

Figure S2.4. Raw solutions chosen for the consensus cluster at different "maxclust" values. Panels (a), (b), and (c) show raw solutions falling into the consensus cluster given a total cluster number ("maxclust" value) of 5, 50, and 250, respectively, for the prototypic system described in Additional file 3. Panel (d) shows the averaged consensus cluster for the chosen "maxclust" value of 50.

Figure S2.1

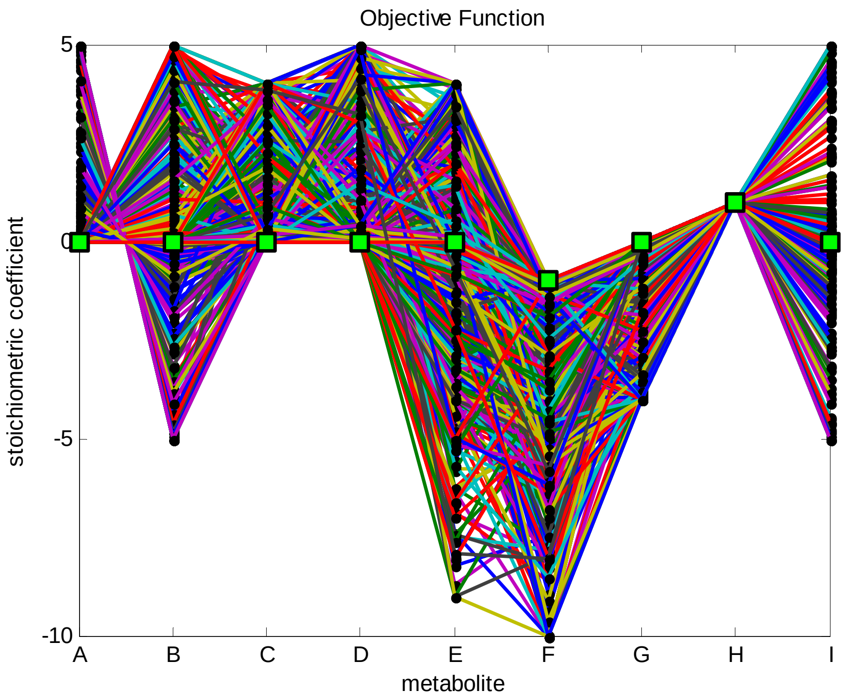

Objective Function
stoichiometric coefficient
metabolite
5
0
-5
-10
A
B
C
D
E
F
G
H
I

Figure S2.2

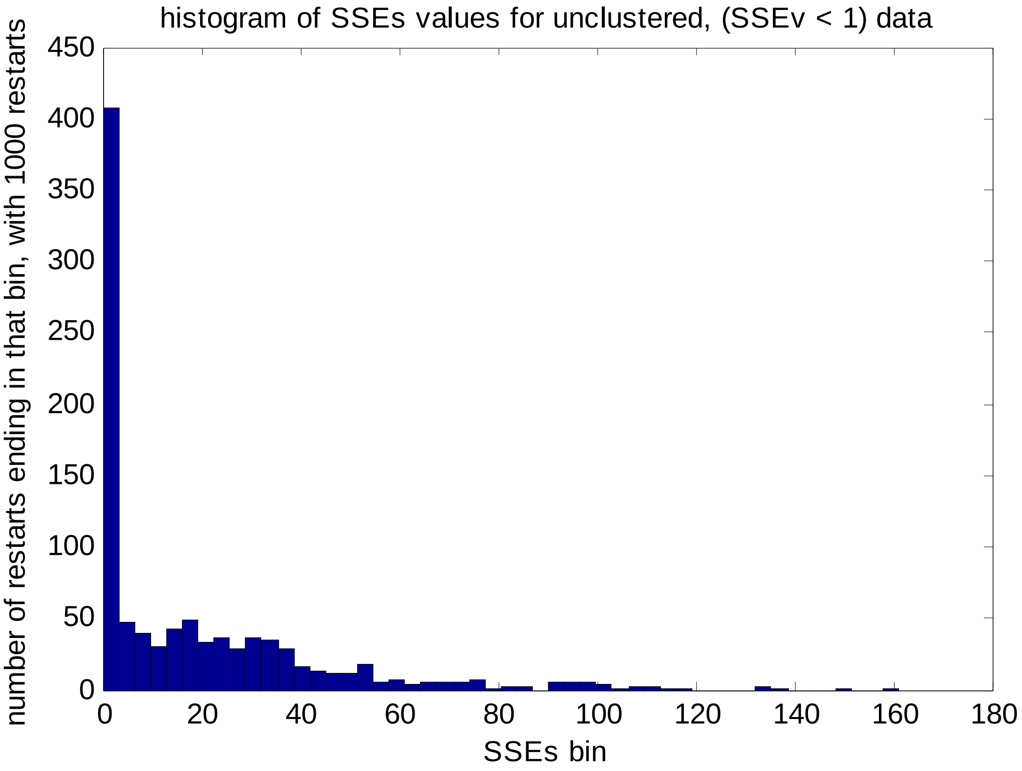
histogram of SSEs values for unclustered, (SSEv < 1) data
number of restarts ending in that bin, with 1000 restarts
450
400
350
300
250
200
150
100
50
0
0
20
40
60
80
100
120
140
160
180
SSEs bin

Figure S2.3

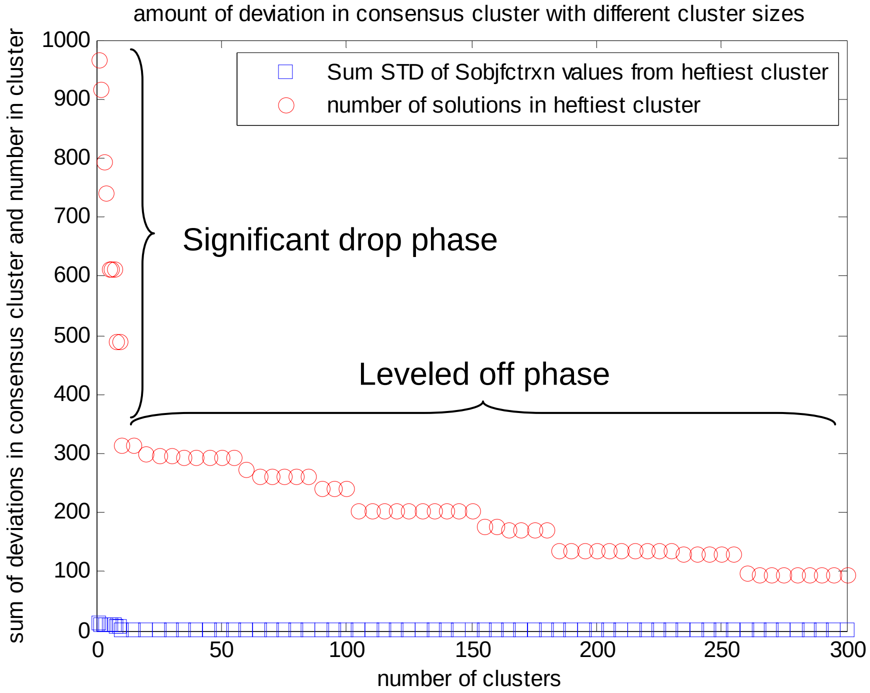
amount of deviation in consensus cluster with different cluster sizes
Sum STD of Sobjfctrxn values from heftiest cluster
number of solutions in heftiest cluster
Significant drop phase
Leveled off phase
sum of deviations in consensus cluster and number in cluster
1000
900
800
700
600
500
400
300
200
100
0
0
50
100
150
200
250
300
number of clusters

Figure S2.4

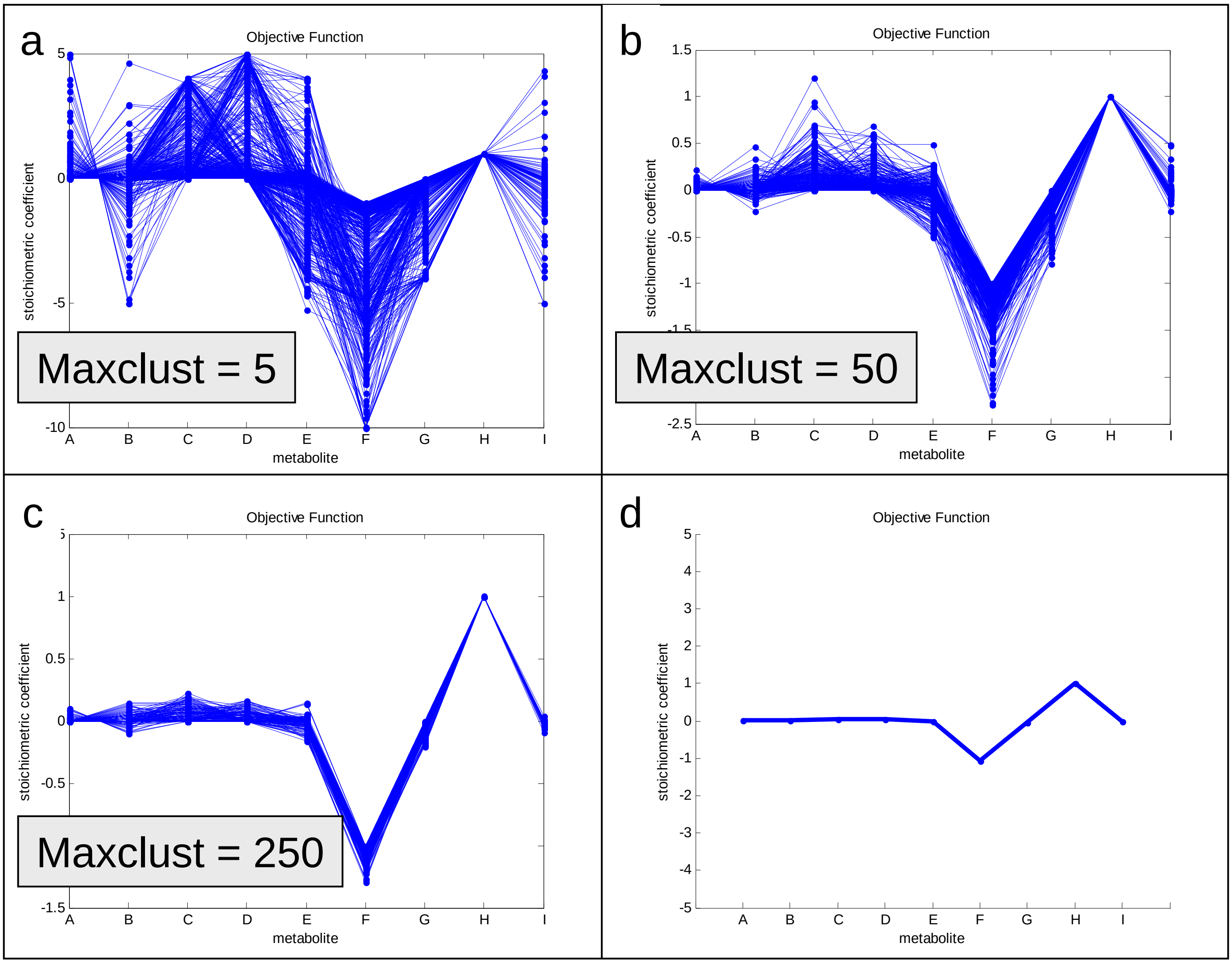
a
Objective Function
stoichiometric coefficient
metabolite
A
B
C
D
E
F
G
H
I
5
0
-5
-10
Maxclust = 5
b
Objective Function
1.5
1
0.5
0
-0.5
-1
-2.5
Maxclust = 50
c
Objective Function
1
0.5
0
-0.5
-1.5
Maxclust = 250
d
Objective Function
5
4
3
2
1
0
-1
-2
-3
-4
-5
