# Supplementary material for: Predicting biological system objectives de novo from internal state measurements
Source: BMC Bioinformatics. 2008 Jan 24;9:43. doi: 10.1186/1471-2105-9-43 (PMC2258290; doi:10.1186/1471-2105-9-43)
Supplement: Additional File 3 — Evaluating data integration in BOSS. BOSS is applied to a prototypic system designed to illustrate the ability of the framework to infer an objective function by synthesizing information from multiple experimental conditions. [file 1471-2105-9-43-S3.pdf]

ADDITIONAL FILE 3: EVALUATING DATA INTEGRATION IN BOSS

Due to experimental challenges, most experimental data sets are comprised of a subset of metabolic fluxes over multiple conditions rather than all metabolic fluxes for a given experimental condition [1, 2]. To investigate the ability of **BOSS** to synthesize information from multiple experimental conditions beyond the *S. cerevisiae* central metabolic network, a prototypic system was constructed that requires data from multiple single-gene knockouts to unambiguously define an objective reaction. (Note that, although the prototype was constructed to evaluate knockout data, the importance of this experiment was to evaluate whether **BOSS** can integrate multiple conditions at once, not necessarily knockout data itself.)

Methods

This prototypic system, comprised of 9 metabolites and 13 reactions, is shown in Figure S3.1(a), and the reactions are listed in Figure S3.1(b). Fluxes for reactions 2, 3, 4, 7, and 9 are considered to be known through *in vivo* experimentation (as illustrated by the dashed blue squares), and reaction 1 is the objective reaction (as illustrated by the orange arrow). A flux distribution for the "wild-type" network (abbreviated WT) was generated using FBA and maximizing for v_1. Additionally, we generated flux distributions for two single-gene knockout conditions, namely the knockout of reaction 7 (v_7 knockout or KO-7) and the knockout of reaction 9 (v_9 knockout or KO-9) by maximizing for v_1. These flux distributions are summarized in Figure S3.1(b) and were inputted into **BOSS** as "experimental" flux data sets. (Note that, although *in silico* flux data were inputted into **BOSS**, the goal of this study was to validate the functionality of **BOSS** on multiple-condition data in a simple system. Future studies can apply **BOSS** to actual biological systems with the types of experimental data sets simulated here.) The objective reaction (v_1) was then removed from each system before **BOSS** was applied, as described below. Due to topological properties built into the prototype, the data from the v_7 knockout by itself is consistent with four possible objective reactions (as illustrated by the cyan arrows on Figure S3.1(c)), while the data from the v_9 knockout by itself is consistent with three possible objective reactions (Figure S3.1(d)). Only one data-consistent objective reaction overlaps between the wild-type, v_7 knockout, and v_9 knockout flux data, i.e., the true objective reaction for the system,

v_1 (as illustrated by the orange arrow in Figures S3.1(a), S3.1(c), and S3.1(d)). Thus, the prototype provides a test for situations in which flux data from multiple experimental conditions (e.g., multiple single-gene knockouts, multiple-gene knockouts, differing substrates, and uptake limitations) are required in order to ascertain the correct objective reaction. No one knockout case, nor the wild-type case alone, unambiguously points to the correct objective reaction.

Results and Conclusions

FBA was used to optimize the wild-type prototypic system as well as two single-gene knockout variants of the system for a pre-determined objective reaction. For each of these three experimental conditions, only a subset of the flux data was kept and inputted into the framework in an attempt to simulate the incomplete nature of experimental flux data. (Note that, as previously stated, although *in silico* flux data were inputted into **BOSS**, the goal of this study was concept/technique validation.) This subset, shown in dashed boxes in Figure S3.1(a), includes reactions 2, 3, 4, 7, and 9. The objective functions that were identified by the framework are shown in Figure S3.2. Specifically, Figure S3.2 illustrates four knockout cases: the objective reaction coefficients for the 9 metabolites when only a subset of the wild-type (WT) experimental flux data is specified (Figure S3.2(a)); when subsets of the WT data and of the reaction 7 knockout (KO-7) variant flux data are specified together (Figure S3.2(b)); when subsets of the WT flux data and reaction 9 knockout (KO-9) flux data are specified together (Figure S3.2(c)); and when subsets of the WT flux data, the KO-7 flux data, and the KO-9 flux data are specified together (Figure S3.2(d)). Interestingly, the final objective functions for these four sets of data clumped into two general shapes.

Taking WT (Figure S3.2(a)) as well as the WT coupled with KO-9 data (Figure S3.2(c)), the consensus objective reaction closely approximates an exchange of one unit of metabolite C for one unit each of metabolites G and H (see inset of Figures S3.2(a) and S3.2(b)). There are two features of this consensus reaction that are of particular interest. First, the reaction is approximately mass balanced. The objective reaction is improperly mass-balanced by 0.04 mass units, which is 4 percent of the smallest massed components of the system. This mass balancing occurs despite the fact that metabolite masses are not explicitly tracked by **BOSS**, and the error

arises due to the averaging of slightly different, more accurately mass-balanced objective reactions in the consensus cluster. Second, the putative objective reaction makes logical sense since, when provided with only the WT data (Figure S3.1(b)), there is no way for **BOSS** to know whether reactions 8, 11, or 12 have any flux through them. Thus, the framework can predict an objective reaction that trumps those three reactions, taking metabolite *C* straight to metabolites *G* and *H*. With WT and KO-9, the knockout data does not add significantly to what the WT alone tells **BOSS** about the system. This is due to the fact that in the WT case, all flux preferentially flows through reaction 7 instead of reaction 5 or reaction 9, due to the greater yield of final product through that path. Since reaction 7 contains all outflow from metabolite *A* in the WT case, reaction 9 is effectively knocked out. This explains why adding KO-9 to the WT data does not significantly alter the objective reaction.

More accurate objective reactions are obtained from cases including KO-7 data in addition to the WT data (Figures S3.2(b) and S3.2(d). KO-7 forces flux through a different pathway than it would normally go, thereby altering the entire flux distribution through the system and lending insight to the **BOSS** framework as to how the objective function must be structured. The KO-7 data eliminate metabolites *E* and *C* as possible precursors to *H*, since the data indicate that flux can flow through metabolite *H* without any flux through metabolites *C* or *E*. The results for both trials where KO-7 data were included (Figures S3.2(b) and S3.2(d)) approximate the original objective reaction, indicating that the **BOSS** framework is capable of successfully integrating information from multiple knockouts in predicting a most-likely objective.

References

1. Li M, Ho PY, Yao S, Shimizu K: **Effect of lpdA gene knockout on the metabolism in Escherichia coli based on enzyme activities, intracellular metabolite concentrations and metabolic flux analysis by 13C-labeling experiments**. *J Biotechnol* 2006, **122**(2):254-266.
2. Emmerling M, Dauner M, Ponti A, Fiaux J, Hochuli M, Szyperski T, Wuthrich K, Bailey JE, Sauer U: **Metabolic flux responses to pyruvate kinase knockout in Escherichia coli**. *J Bacteriol* 2002, **184**(1):152-164.

Figure captions

Figure S3.1. The prototypic system. In panel (a), a map of the prototypic system designed for evaluating the framework's ability to synthesize information from multiple experimental conditions is shown. This system consists of 9 metabolites depicted as green circles and 13 reactions depicted as black arrows. The objective reaction for this system is reaction 1, and it is drawn as an orange arrow. For the purposes of evaluating our framework on this system, we assumed that the fluxes for reactions 2, 3, 4, 7, and 9 are known through *in vivo* experimentation; these reactions are highlighted by the highlighted blue boxes. Panel (b) lists the reactions for the network and the "experimental" fluxes (generated via FBA) for the wild-type system and the variants wherein reactions 7 and 9 are knocked out of the system. The rows shaded in yellow highlight the fluxes across these three conditions for the reactions that we assumed we would know through *in vivo* experimentation. Panels (c) and (d) illustrate the system after reactions 7 and 9 are knocked out (v_7 knockout or KO-7 and v_9 knockout or KO-9), as shown by the shaded arrows for v_7 and v_9, respectively. The flux data from each of these knockouts coupled with the network's topological features enable three possible objective reactions, and these are depicted by the cyan arrows. Ultimately, when the flux data from the wild-type in panel (a), the v_7 knockout in panel (c), and the v_9 knockout in panel (d) are together evaluated by our framework, only one reaction depicted as an orange arrow in panels (a), (c), and (d) is a valid objective reaction for the system.

Figure S3.2. The predicted objective reactions for the prototypic system for multiple experimental conditions. The objective reactions predicted by our framework are illustrated. Panel (a) depicts the result when only a subset of the wild-type flux data is inputted into the framework. Panel (b) depicts the result when subsets of the wild-type flux data and the v_7 knockout variant flux data are inputted into the framework. Panel (c) depicts the result when subsets of the wild-type flux data and the v_9 knockout variant flux data are inputted into the framework. Panel (d) depicts the result when subsets of the wild-type flux data, the v_7 knockout variant flux data, and the v_9 knockout variant flux data are inputted into the framework. The predetermined objective reaction artificially incorporated into this system, i.e., the conversion of F to H, is best reproduced by the last case shown in panel (d). The insets of the figures illustrate the **BOSS**-derived objective reactions overlaid on top of the network topology.

Figure S3.1

a

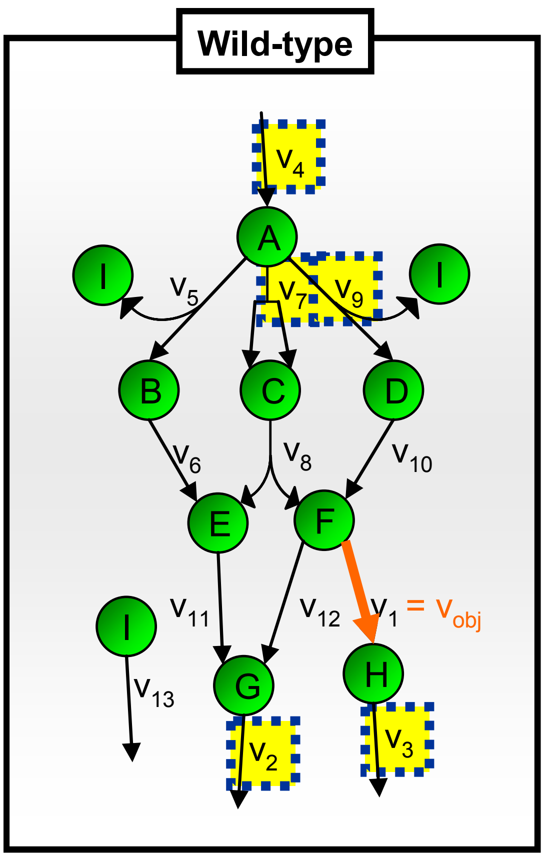

b

Rxn No.	Reaction	Experimental Fluxes		
		WT	KO-7	KO-9
1	-1 F +1 H	20	10	20
2	-1 G	20	0	20
3	-1 H	20	10	20
4	+1 A	10	10	10
5	-1 A +1 I + 1B	0	0	0
6	-1 B +1 E	0	0	0
7	-1 A +2 C	10	0	10
8	-1 C +1 E +1 F	20	0	20
9	-1 A +1 D +1 I	0	10	0
10	-1 D +1 F	0	10	0
11	-1 E +1 G	20	0	20
12	-1 F +1 G	0	0	0
13	-1 I	0	10	0

c

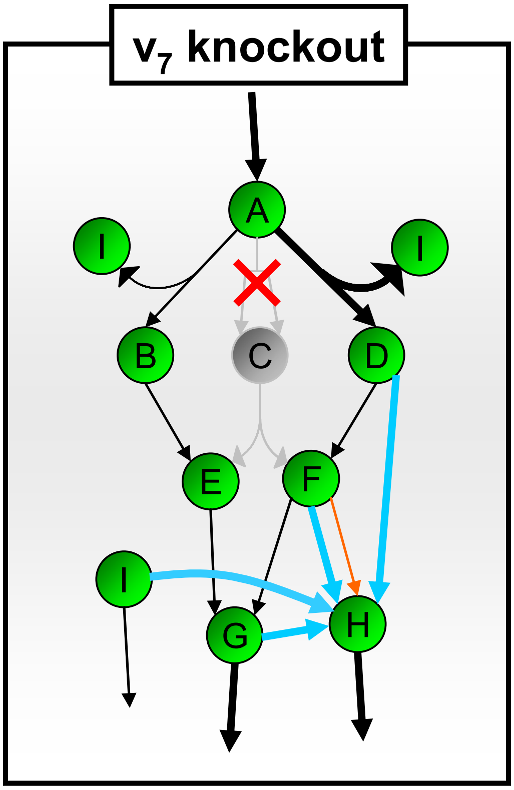

d

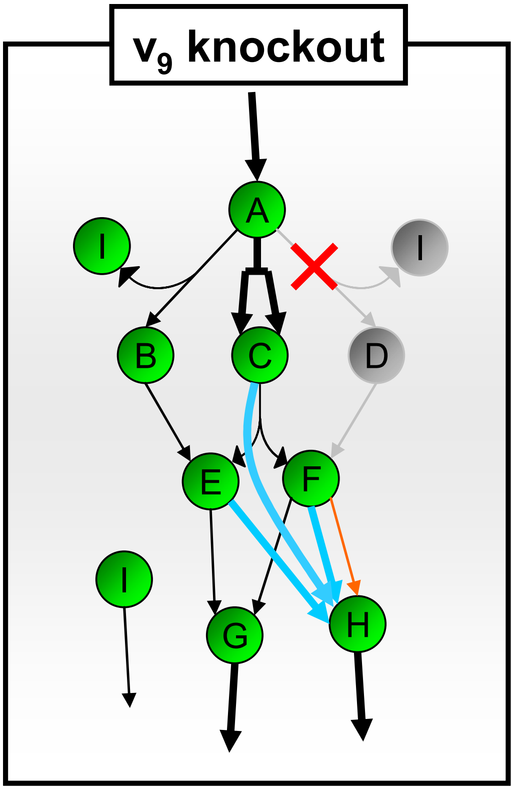

Figure S3.2

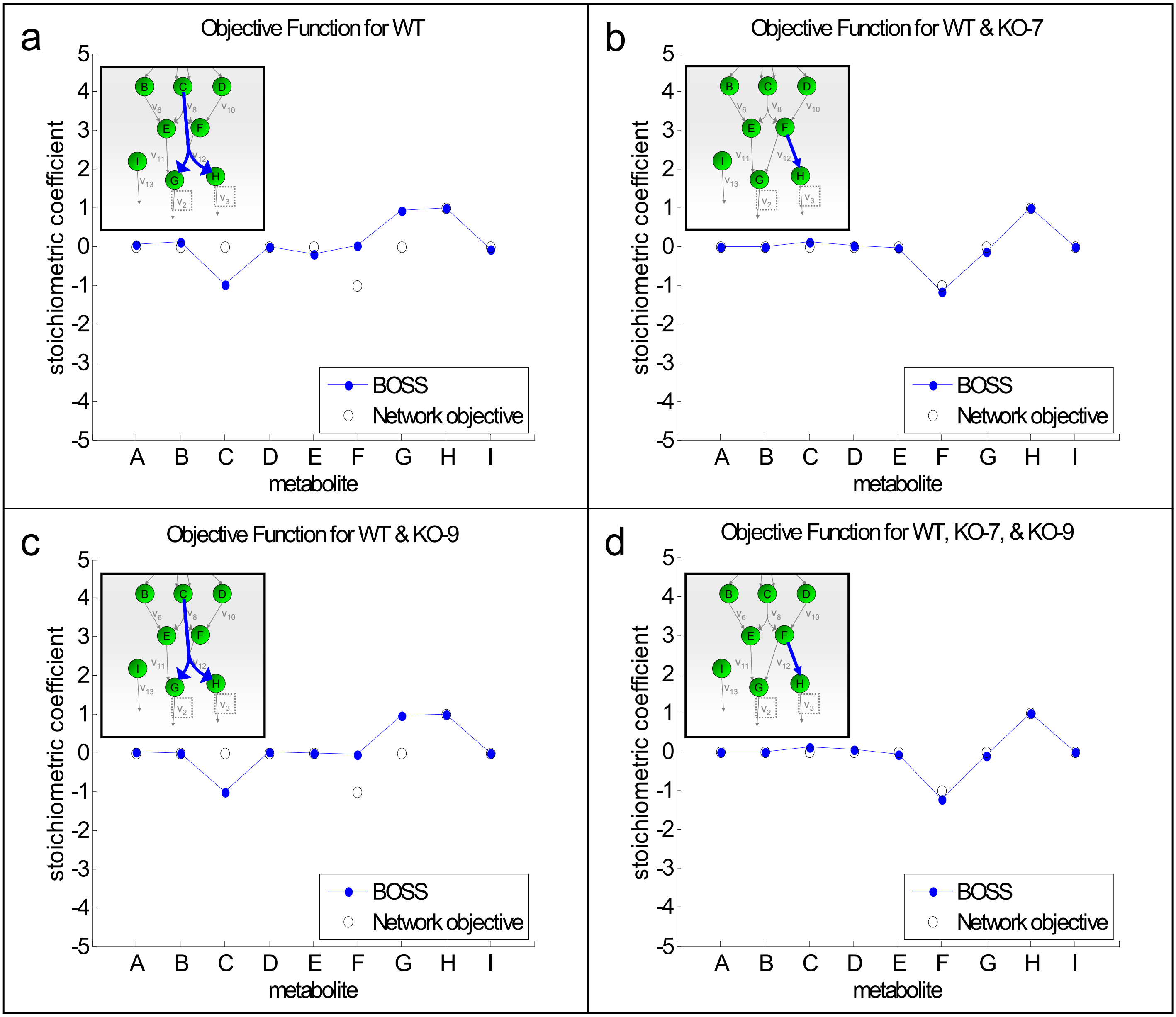
a
Objective Function for WT
b
Objective Function for WT & KO-7
c
Objective Function for WT & KO-9
d
Objective Function for WT, KO-7, & KO-9
stoichiometric coefficient
metabolite
A B C D E F G H I
5 4 3 2 1 0 -1 -2 -3 -4 -5
BOSS
Network objective
